# Coping with an Uncertain or Poor Cancer Prognosis as an Adolescent or Young Adult: A Cross-Sectional Cluster Analysis

**DOI:** 10.3390/curroncol33070376

**Published:** 2026-06-23

**Authors:** Milou J. P. Reuvers, Winette T. A. van der Graaf, Olga Husson, Leyla Azarang

**Affiliations:** 1 Department of Medical Oncology, Netherlands Cancer Institute—Antoni Van Leeuwenhoek, 1066 CX Amsterdam, The Netherlands; m.reuvers@nki.nl (M.J.P.R.); w.vd.graaf@nki.nl (W.T.A.v.d.G.); l.azarang@nki.nl (L.A.); 2 Department of Medical Oncology, Erasmus MC Cancer Institute, Erasmus University Medical Center, 3015 CP Rotterdam, The Netherlands; 3 Departments of Public Health & Surgical Oncology, Erasmus MC Cancer Institute, Erasmus University Medical Center, 3015 CP Rotterdam, The Netherlands

**Keywords:** adolescents and young adults, oncology, uncertain or poor cancer prognosis, cluster analysis, quality of life

## Abstract

Background: A subgroup of adolescent and young adult patients (AYAs; 18 to 39 years at diagnosis) face an uncertain or poor cancer prognosis (UPCP). Previous qualitative research identified dual coping pathways in this population: engagement in life versus the reality of premature death. This study examines whether similar psychosocial profiles can be identified through quantitative data, aiming to differentiate patient experiences and identify characteristic features of each cluster. Additionally, this study examines the association between cluster membership and social support needs to understand psychosocial disparities. Methods: Eligible participants completed questionnaires assessing physical, psychosocial, and existential outcomes related to their disease and prognosis. An ensemble clustering approach was applied, including evaluation of clustering tendency and multiple algorithms, with stable clusters identified through majority voting. Associations with social support needs were analyzed using Fisher’s exact test. Results: Data from 155 AYAs with a UPCP were included. The mean age at diagnosis was 31.2 years, with glioma (34.8%) and breast cancer (17.4%) as the most common diagnoses. Two distinct clusters were identified: one (22%) characterized by poorer functional outcomes and fewer protective factors (e.g., hope, meaning in life), and another cluster (78%) with better functioning and less frequent needs for social support (*p* < 0.00043). Conclusions: Findings revealed divergent psychosocial profiles within the AYA-UPCP population, highlighting the importance of early identification of vulnerable subgroups. Strengthening protective factors may enhance resilience and reduce unmet support needs. Validation in larger, external datasets is needed to confirm these pathways and guide tailored supportive care strategies.

## Simple Summary

A subgroup of adolescent and young adult patients (AYAs; 18–39 years at diagnosis) face an uncertain or poor cancer prognosis (UPCP), which is associated with substantial psychosocial burden during a key developmental life stage. Previous qualitative research has suggested dual coping pathways in this population, reflecting engagement in life versus awareness of premature mortality. This study explored whether similar patterns could be identified using quantitative data and examined their association with social support needs. Data from 155 AYAs with a UPCP were analyzed using an ensemble clustering approach. Two exploratory subgrouping patterns were identified, primarily reflecting differences in overall psychosocial burden rather than clearly distinct patient types. Patients with lower burden reported fewer social support needs. These findings are clinically relevant as they suggest that psychosocial vulnerability is linked to unmet support needs, supporting the value of routine screening for distress and social functioning in clinical practice. Rather than relying on predefined subgroups, identifying patients with elevated burden may be more effective for targeting supportive care. Further validation in larger longitudinal datasets is needed to confirm these patterns and inform tailored interventions.

## 1. Introduction

Cancer patients encounter practical, social, psychological, and spiritual challenges stemming from their illness, treatment, and prognosis. Coping strategies can help individuals to adjust to the disease, improving their well-being [1,2]. Lazarus defined coping as “the process of managing external and/or internal demands that tax or exceed the resources of the person” [3]. Coping is linked to symptom burden [1] and can be influenced by age; for example, younger patients are more likely to seek social support [4]. For advanced cancer patients, coping is particularly important due to the ongoing unpredictability of the disease trajectory. While coping is often described as dynamic, the extent to which patients adjust their strategies over time may vary. Furthermore, uncertainty related to advanced disease can lead to distress, which coping strategies can alleviate, especially during prolonged uncertainty (e.g., with the emergence of immunotherapy and targeted therapy). Cognitive avoidance may help to avoid weariness and rumination caused by constant worry [5,6].

**Figure 1:**
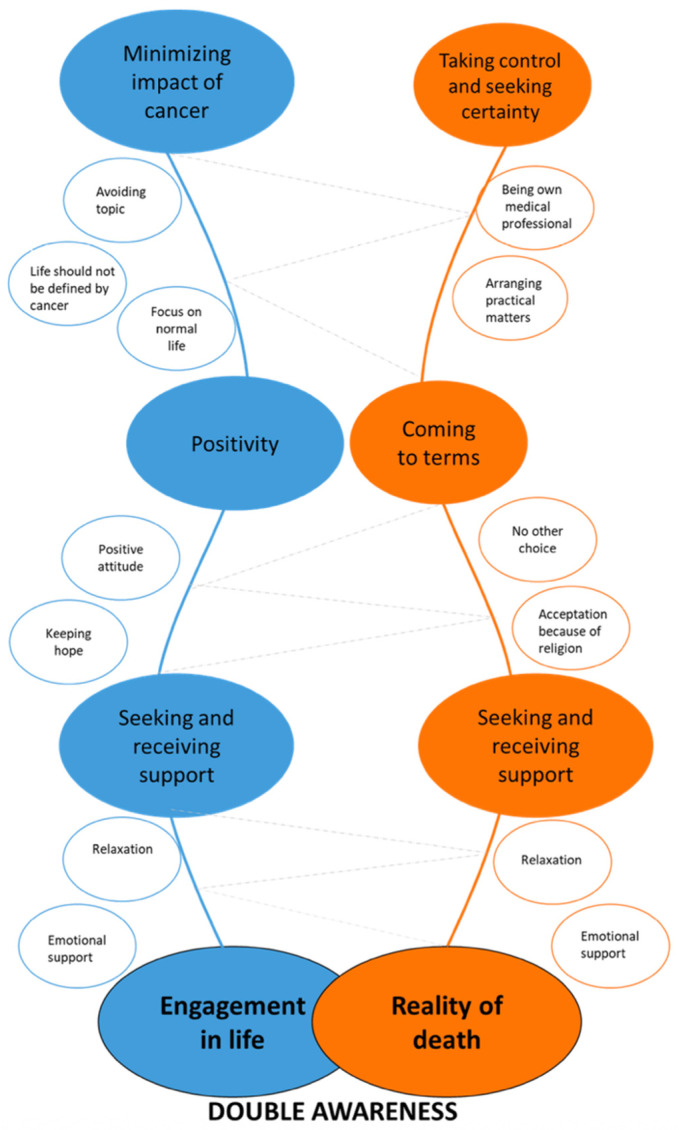
Dual pathways of coping among AYAs with a UPCP [10].

In the Netherlands, approximately 4200 adolescents and young adults (AYAs; 18 to 39 years at diagnosis) are diagnosed with cancer each year [7]. While their relative five-year survival rate is 80–85% [8], some face an uncertain or poor cancer prognosis (UPCP): they are either diagnosed with advanced cancer (e.g., metastatic disease) or lack curative treatment options (e.g., glioma). These patients are likely to die from their disease, though not in the short-term [9]. Our qualitative study showed that AYAs with a UPCP engage in coping strategies that can be divided into dual pathways: engagement in life and/or facing premature death (Figure 1). Patients actively balance normal life and the disease: they strive for normalcy (meaningful activities, maintaining roles) but are sometimes forced to face the impact of disease and prognosis (i.e., awareness of mortality and anticipatory grief) [10]. AYAs with a UPCP tend to follow one of these pathways, although it is naturally possible that they shift between them over the course of their long illness trajectory. Other studies also found that AYAs with advanced cancer experience this dichotomy [11,12]: they appear to be proactive in managing their healthcare but also distance themselves from their disease [11,13]. Social support is vital in both paths [10], yet AYAs feel different from healthy peers, AYAs receiving curative treatment, and older adults with a UPCP [14]. Moreover, psychological distress, physical symptoms, difficulties undertaking usual activities important at AYA age (e.g., employment), and reduced autonomy are associated with social difficulties, with support often decreasing over time [15]. Therefore, social support may also differ between the pathways of AYAs with a UPCP.

Understanding the experiences of advanced cancer patients can inform supportive care interventions aimed at enhancing coping strategies to manage problem-solving abilities related to uncertainty and future worries [1]. AYAs with a UPCP may benefit even more as they severely struggle with future uncertainty due to their developmental life phase [16]. Using adaptive coping strategies has been associated with greater resilience and empowers patients to deal with cancer-related stressors [17,18]. Research among stroke patients suggests that resilience can act as a protective factor for illness uncertainty, by decreasing the impact of suboptimal coping strategies [15]. In AYAs, resilience-focused interventions have been shown to improve health-related quality of life (HRQoL) and lower psychological distress [16,17,19].

Building on previous qualitative studies [10], our study adopts an exploratory approach to examine patterns in patient-reported outcomes among AYAs with a UPCP using a clustering analysis to observational questionnaire data. This approach aims to explore whether the data suggests potential subgroups based on similarities in psychosocial functioning and coping-related variables. The aim is to identify whether there are any natural groups within our study cohort by grouping similar cases, and to assess whether these mirror elements of the pathways defined by Burgers et al. [10]. In this study, we do not attempt to directly quantify coping pathways but explore whether psychosocial profiles show patterns that may relate to these pathways. Assuming the validity of our clustering approach in accurately identifying distinct paths, this study explores whether these profiles differ in reported social support needs. Specifically, we hypothesize that social support needs are significantly associated with these profiles, allowing us to enable targeted support for those who benefit most and provide proactive interventions before profound challenges arise. As this is the first study with quantitative data on AYAs with a UPCP, it addresses an important gap in the current literature.

## 2. Materials and Methods

### 2.1. Study Information

Data from the CORD-AYA study (Balancing on a tightrope—living with an uncertain or poor cancer prognosis as an adolescent or young adult) were used. This is an observational, longitudinal questionnaire study among AYAs with a UPCP, aiming to provide insight into patterns of suffering, challenges and meaningful living, and examining factors associated with these outcomes. Patients complete a questionnaire every three months over two years. This article focusing on the baseline results.

#### 2.1.1. Eligibility Criteria

Burgers et al. [2] have defined AYAs with a UPCP as “those with advanced cancer for which there is no reasonable hope of cure, indicating that they will die prematurely from cancer, but have no immediate threat of death”. This definition has been adjusted for this study, assuming that patients are “likely” to die prematurely because of their disease. This adjustment reflects that new therapies do not allow us to state with certainty that cure is impossible in the long-term.

Patients were eligible if they were diagnosed between the ages of 18 and 39 years and were facing advanced cancer at study participation (e.g., stage IV disease at diagnosis, metastasized disease occurring after diagnosis) or a glioma, had an (estimated) life expectancy of at least twelve months, and were not older than 45 at the time of study enrollment, as some patients mentioned that, beyond this age, they no longer identified as an AYA. Patients could participate at any time after diagnosis. Exclusion criteria included the inability to read Dutch or English sufficiently, or any cognitive or practical problems interfering with completing questionnaires.

#### 2.1.2. Procedure

Ten Dutch hospitals participated in the CORD-AYA study: four academic hospitals and six teaching hospitals. Patients were invited by the researcher when their treating physician perceived them as eligible based on the inclusion criteria. Additionally, patients could be invited via word-of-mouth (e.g., via social media or patient organizations). An e-mail was sent out with study information and the informed consent form. If a patient did not respond within 1–2 weeks, a reminder was sent. If there was still no response, the researcher called or emailed the patient once more. After informed consent was signed, patients could complete the questionnaire either on paper or online via Castor. The study was approved by the Institutional Reviewing Board of the Antoni van Leeuwenhoek-Netherlands Cancer Institute (IRBd22-344).

### 2.2. Summary of Measures

Here, a summary of measures is provided. The complete information of measures is reported in Appendix A.

Sociodemographic and clinical characteristics were self-reported by patients, including questions on their year of birth, partner status, living situation, educational level, work status, age at diagnosis, cancer type and treatment, the aim of their treatment, and additional support they received.

Problems and needs were measured using the Problems and Needs in Palliative Care Questionnaire (PNPC) [20]. Quality of life was measured using the EORTC QLQ-C30 [21,22]. Anxiety was measured using the General Anxiety Disorder (GAD-7) questionnaire [23]. Depression was measured using the Patient Health Questionnaire (PHQ-9) [24]. Demoralization was measured with the Demoralization Scale (DS), a 24-item questionnaire on existential distress in advanced cancer patients [25]. Meaning and purpose was measured using the PROMIS Meaning and Purpose scale [26]. Hope was measured using a self-developed item based on our previous qualitative work [27]: “I believe that something will happen that will cure me”. Life urgency was also measured using a self-developed item based on our previous qualitative work [27]: “I feel the urge to do as many things as possible in a short amount of time because of my uncertain or poor prognosis”. Goal adjustment was measured using the Goal Adjustment Scale (GAS) [28]. Illness uncertainty was measured using the Mishel Uncertainty in Illness Scale (MUIS-A) [29]. Self-efficacy was assessed using the Self-Efficacy for Managing Chronic Disease (SEMCD) [30]. Coping was measured using the BRIEF-COPE questionnaire [31]. Resilience was examined using the 10-item Connor Davidson Resilience Scale (CD-RISC-10) [32]. Social support was assessed using an adapted version of the Duke-UNC Functional Social Support Questionnaire (FSSQ). This modified version includes five items, with three general items combined into one focusing on discussing the disease and related problems with others [33].

### 2.3. Data Analysis

#### 2.3.1. Descriptive Statistics

Sociodemographic and clinical data were analyzed using frequencies and descriptives. Scale scores were calculated and reported using means and standard deviations. SPSS version 30.0 was used for descriptive statistics.

#### 2.3.2. Cluster Analysis and Reproducibility

In order to explore potential profiles among CORD-AYA patients, we considered two datasets: one with complete cases (155 patients with 226 complete scales), and another including both complete scales and scales with less than 5% missing information (including 143 patients and 408 complete scales). To assess the clustering tendency of each dataset, we conducted Hopkins statistic test [34], using 0.5 as the threshold: if less than 0.50, it is unlikely that the dataset has statistically significant clusters. In other words, if the value of Hopkins statistic is at least larger than 0.70, we can conclude that the dataset is significantly clusterable. The Hopkins statistic was 0.59 for the complete dataset and 0.58 for the dataset with less than 5% missing data. Although these values do not provide strong evidence that either dataset is significantly clusterable, they also do not suggest a complete absence of clustering tendency. This implies there may be some degree of underlying structure among the CORD-AYA patients, albeit not particularly strong—possibly due to the relatively small sample size. This weak clustering tendency is common in psychosocial datasets and therefore supports the usage of an ensemble approach. Despite the modest clustering indication, we proceeded with cluster analysis using only the dataset with complete items (all 155 patients and 226 fully completed items). This dataset was selected because it includes all participants, has a slightly higher Hopkins statistic, and exhibits lower multicollinearity. Although the Hopkins statistic indicated only modest clusterability (0.58–0.59), this is common in psychosocial datasets with high dimensionality and moderate sample sizes. The ensemble approach was therefore chosen to maximize robustness. The significant SigClust result (*p* < 0.001) supports that the identified clusters represent non-random structure.

To address the moderate clusterability tendency of the CORD-AYA cohort, we applied an ensemble clustering framework to conduct the cluster analysis. This framework allowed us to select an ensemble from a diverse set of clustering algorithms. Below, we outline our clustering procedure step by step. First, we considered an ensemble of the following algorithms: “K-Means”, “Partition Around Medoids (PAM)”, “Hierarchical Agglomerative”, “Hierarchical Divisive”, and a “Self-Organizing Map (SOM) with Hierarchical Clustering”. The distance functions for PAM and both hierarchical models are “manhattan” and “euclidean”. Second, we considered three different numbers of clusters (2, 3, and 4). Third, for each algorithm and each cluster size, we performed subsampling using 80% of the data, repeated this five times and applied the algorithms to each subsample. Every algorithm with three different numbers of clusters was applied to 5 subsets of the data, each consisting of 80% of the original observations. Because of subsampling (not every observation is included in each clustering), we “completed” the data using k-nearest neighbor.

Fourth, to assess the cluster size with the greatest clustering stability, we calculated the proportion of ambiguous clusters. Fifth, we used several internal clustering validation indices (criteria) to evaluate the clustering algorithms. These indices assess compactness (how similar the objects are within a cluster), separation (how distinct the objects are across different clusters), and robustness (how consistently the clusters can be reproduced in other datasets). We used the following indices: “Dunn”, “PBM”, “Silhouette”, “Davies Bouldin”, “SD_Dis”, “Ray_Turi”, “Compactness” and “Connectivity”. The algorithms with small “Davies Bouldin”, “SD_Dis”, “Ray_Turi”, “Compactness”, and “Connectivity” indices and large “Dunn”, “PBM”, “Silhouette” indices were selected for inclusion in the cluster ensemble. Accordingly, the algorithms with poorer performance were excluded from the cluster ensemble. The variability across internal validity indices reflects the absence of a single dominant clustering solution and supports the exploratory nature of the analysis. Sixth, we applied a “Majority Voting” method for pooling the results of the remaining algorithms, where the final clusters were determined based on the majority consensus among the optimal algorithms. Finally, we applied “SigClust” approach to test whether the concluded set of clusters really existed or there were no cluster at all.

Additionally, we performed Fisher’s exact test to determine the association between the clustered paths and patients’ self-reported need for social support, using an item of the PNPC (i.e., experiencing too little support by others). The answering options were no (no need for additional support), as much as now (receiving sufficient support), or yes (a need for more support). The need for social support is an external item (i.e., an item that is not included in cluster analysis), and there were no other internal items related to social support. Cluster analysis and Fisher’s exact test were performed in R version 4.5.1 (R Development Core Team and the R Foundation for Statistical Computing), integrating software from open-source packages, including “factoextra”, “diceR” [35], “sigclust” and packages from “tidyverse” [36], including “dplyr”, “tidyr”, and “ggplot2”. A *p*-value < 0.05 was considered statistically significant. For further details on the statistical analysis are provided in the Supplementary Materials files.

## 3. Results

### 3.1. Descriptive Analysis

A total of 155 AYAs with a UPCP participated in this study. The mean age at diagnosis was 31.2 (±5.5), and the most common tumor types were glioma (34.8%) and breast cancer (17.4%). Cluster “1” had a mean age at diagnosis of 30.2 (±4.8), and Cluster “2” 31.5 (±5.7). Sociodemographic and clinical factors of both clusters are described in Table 1. The full questionnaire contained 422 items, 408 items less than 5% missing information, and 226 items were complete (Table 2). External factors have been reported in Appendix B.

### 3.2. Cluster Analysis

The ensemble clustering method resulted in the selection of two as the consensus cluster size (i.e., number of clusters), as this corresponded to the smallest proportion of ambiguous clusters, indicating the most stable set. Once the number of clusters was set to two, we evaluated the performance of each algorithm. The internal validity indices suggested the inclusion of the following algorithms in the cluster ensemble: “Hierarchical Agglomerative” with “manhattan”, “Hierarchical Divisive” with “euclidean”, and “Hierarchical Divisive” with “manhattan” distance measure. Therefore, the final assignment of patients into two clusters was derived from the results of the three algorithms.

**Figure 2:**
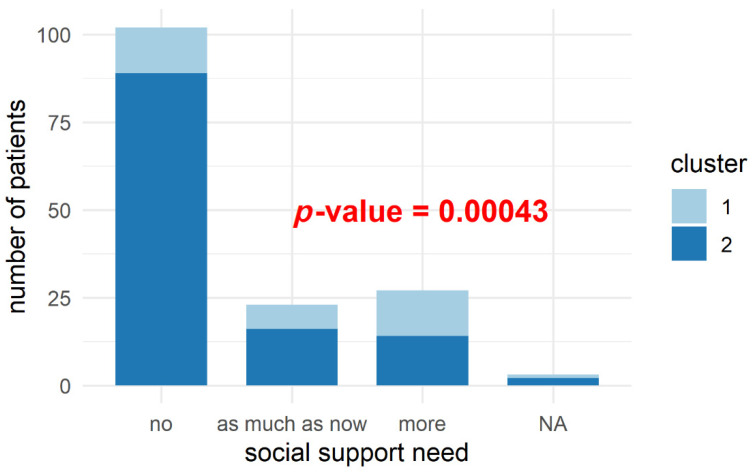
Association between patient clusters and their need for social support.

Furthermore, according to the *p*-value obtained from the “SigClust” test, our clustering result was statistically significant (*p* < 0.001), indicating sufficient evidence to conclude the existence of two explorative clusters in our dataset. Cluster “1” comprised 21.9% of patients, whereas Cluster “2” included 78.1%. The resulting two-cluster solution was asymmetric, with Cluster 1 comprising a relatively small proportion of the sample. This imbalance may reflect either meaningful heterogeneity within a minority subgroup or a subset of patients with more extreme psychosocial profiles. Given the exploratory nature of the analysis, both interpretations remain plausible and should be interpreted with caution. The overarching pattern indicated that individuals in Cluster “1” showed a pattern of higher psychosocial burden based on descriptive indications compared with those in Cluster “2”. They showed higher levels of psychosocial burden (anxiety, depression, demoralization) and fewer personal resources that could support coping (self-efficacy, resilience, social support). However, coping did not appear to differ between the clusters, with only avoidant coping being more common among those in Cluster “1”, who also reported greater life urgency and a lower sense of meaning. Our Fisher’s exact test revealed a statistically significant association between patients’ pathways and their need for social support. As shown in Figure 2, the majority of patients who did not require additional social support tended to be on the better pathway (Cluster “2”), whereas the number of patients requiring more social support was similar for both pathways.

## 4. Discussion

This study explored psychosocial profiles within a sample of AYAs with a UPCP using a clustering analysis, suggesting the presence of two clusters that reflect differences in overall psychosocial burden. In line with the work of Burgers et al., [10,16], our results suggest heterogeneity in how AYAs with a UPCP experience and cope with their disease. However, our quantitative analysis did not identify their exact pathways [10]. One cluster consistently reported poorer outcomes, suggesting a greater overall psychosocial burden and impact of the disease. Although some characteristics in Cluster “1” resemble elements described in the dual-pathway model (lower meaning in life, stronger focus on treatment), the clusters should not be interpreted as coping pathways. The absence of clear differences in coping strategies may indicate that AYAs use a range of coping strategies regardless of their distress level, or that it is more dynamic and context-dependent than can be captured at a single time point. Longitudinal research is needed to further examine how these processes evolve over time and whether patterns shift, as suggested in prior work [10]. However, supporting these patients in meaningful living and open communication about their disease can lower existential distress, increase their double awareness, improving HRQoL and adaptive coping [37]. This study provides the first quantitative evidence identifying distinct psychosocial burden profiles among AYAs with a UPCP, demonstrating their association with social support needs and thereby extending prior qualitative work.

The exploratory design of this study did not allow for determining statistical differences between clusters. Although true labels were unavailable, our ensemble clustering method effectively identified two patient pathways. A significant association emerged between these pathways and patients’ need for social support. Patients with lower psychosocial burden were less likely to report a need for social support. Social functioning is a protective factor for the well-being of individuals with long-term disease [38], and even more so for AYAs, for whom social connections are a core aspect of identity and development [39,40]. Among AYAs with a UPCP, social support has been shown to be important as many feel lonely or isolated [16]. Moreover, impaired physical health and negative thoughts are associated with reduced social functioning among AYAs [15,41], and social support is related to increased emotional adjustment to disease [42]. This highlights social support as a potential target for supportive care interventions, particularly for those in Cluster “1”. It is crucial that the healthcare team (e.g., nurses, social workers) assess social networks and support among AYAs with a UPCP early on in the disease trajectory, to ensure that patients receive adequate support or can be referred to peer support initiatives [8,39,43] or communication training with their loved ones [39,40]. This can reduce healthcare costs, as reduced social support is often linked to more healthcare utilization and hospitalization [41].

Future work should validate our clusters using external datasets with larger sample sizes to strengthen the findings’ generalizability. A key limitation of this study is the high-dimensional nature of the dataset relative to the sample size (*n* = 155, *p* = 226), which may affect the stability of distance-based clustering methods and increase the risk of unstable solutions. In addition, exploratory analyses, including the Hopkins statistic (0.59), suggest that the dataset does not exhibit strong or clearly separable clustering structure, but rather weak and diffuse patterns of variation. This indicates that any subgroup structure is likely subtle and should be interpreted cautiously. Furthermore, variability across internal validity indices highlights the absence of a single dominant clustering solution, which is not uncommon in high-dimensional and heterogeneous clinical data. Although an ensemble clustering framework with resampling and consensus aggregation was used to improve robustness, the identified clusters should be regarded as exploratory and hypothesis-generating rather than definitive patient subgroups. Overall, these findings reflect the complexity and heterogeneity of psychosocial symptom patterns in this population and underscore the need for cautious interpretation and future validation in independent cohorts. As this study employed an exploratory unsupervised clustering approach, no formal power calculation for subgroup detection was available. Instead, the adequacy of the sample size was evaluated through cluster stability and validation-based criteria, including resampling procedures, internal validity indices, and SigClust testing. Nevertheless, the relatively limited sample size in relation to the number of variables remains a methodological constraint and warrants cautious interpretation of the identified clusters. The present study is part of a larger longitudinal cohort study, for which a priori calculations indicated that at least 100 participants were required; the current sample exceeded this target.

In addition, incorporating clinical differences (e.g., tumor types, treatment phase) is important, as these may affect patients’ coping strategies and overall well-being, and may help explain the psychosocial variation observed between the clusters. Due to a lack of clinical data, we were unable to conduct these analyses. Moreover, disease stage may have impacted their responses and should be considered. Longitudinal research is particularly important to capture the dynamic nature of coping and to examine how patients adjust over time, including potential shifts in psychosocial functioning and support needs [44]. Finally, further exploration of factors such as illness uncertainty, resilience and demoralization may contribute to a more person-centered view of patients’ needs. This may help to inform timely and tailored interventions that strengthen coping strategies. In this context, a flexible coping style may be most beneficial. Research has shown that AYAs often employ various coping strategies during their disease trajectory, which evolve through personal experience and supportive resources. This shows a promising opportunity to actively assist AYAs in developing and refining coping techniques they can draw upon when needed [2]. Moreover, time since diagnosis should be taken into account, as this can significantly affect one’s coping strategies and adaptation to the disease.

This study provides the first quantitative, data-driven explorations of psychosocial functioning in AYAs with a UPCP, paving the way for more individualized supportive care and moving away from a one-size-fits-all approach. However, since our previous analysis showed that AYAs with a UPCP report impaired HRQoL compared to a healthy control group [45], all AYAs with a UPCP require comprehensive screening. Although Cluster “2” seems to exhibit relatively better outcomes, their more favorable status should not overshadow the challenges they may still face or the support they may require. Our findings also encourage multidisciplinary collaborations between medical specialists, psychologists, social workers and other paramedics, ensuring that both psychosocial and existential needs are met. Such collaborations are implemented in AYA-care through the formation of AYA-teams in various Dutch hospitals, which can facilitate this screening. The AYA-anamnesis, an AYA-specific questionnaire used in Dutch healthcare, is being used to discuss age-specific topics and supportive care needs and can be used to further investigate psychosocial burden among these patients. Finally, it is crucial to help patients understand their situation and regain a sense of normalcy in their lives, as this can support everyday coping among patients with an uncertain prognosis [46,47].

## 5. Conclusions

This study identified two exploratory clusters within a sample of AYAs with a UPCP. Patients in Cluster “1” exhibited poorer psychosocial functioning and fewer protective resources compared to those in Cluster “2”, demonstrating heterogeneity in the impact of a UPCP. Notably, the reduced need for social support in Cluster “2” suggests the importance of support to cope with the disease and shows a potential target for supportive care interventions. These findings support the need for tailored supportive care approaches that focus on identifying and addressing elevated distress, while recognizing that all AYAs with a UPCP may experience significant challenges. Future research should prioritize larger samples and adopt a longitudinal approach to better understand coping mechanisms over time, while taking clinical characteristics into account for more effective supportive care interventions.

## Figures and Tables

**Table 1:** Sociodemographic and clinical factors of AYAs with a UPCP (*n* = 155), cluster 1 (*n* = 34) and cluster 2 (*n* = 121).

	Total N (%)	Cluster 1 N (%)	Cluster 2 N (%)
Sex			
Female	89 (57.4)	27 (79.4)	62 (51.2)
Male	65 (41.9)	7 (20.6)	58 (47.9)
Non-binary	1 (0.6)	0 (0.0)	1 (0.8)
Partner			
Yes	124 (80.0)	24 (70.6)	100 (82.6)
No	31 (20.0)	10 (29.4)	21 (17.4)
Living situation			
With partner and children	76 (49.0)	15 (43.1)	61 (50.4)
With partner	42 (27.1)	6 (17.6)	36 (29.8)
Alone	22 (14.3)	9 (26.5)	13 (10.7)
With parent(s)	9 (5.8)	1 (2.9)	8 (6.6)
With roommates/friends	6 (3.9)	3 (8.8)	3 (2.5)
Educational level			
Secondary or less	8 (5.2)	4 (11.8)	4 (3.3)
Secondary vocational	44 (28.4)	10 (29.4)	34 (28.1)
Applied university	59 (38.1)	11 (32.4)	48 (39.7)
University	44 (28.4)	9 (26.5)	35 (28.9)
Occupational status *			
Part-time	40 (25.8)	8 (23.5)	32 (26.4)
Full-time	43 (27.7)	3 (8.8)	40 (33.1)
(Partly) disabled	38 (24.5)	12 (35.3)	26 (21.5)
On sick leave	37 (23.9)	9 (26.5)	28 (23.1)
Unemployed	15 (9.7)	5 (14.7)	10 (8.3)
Student	8 (5.2)	1 (2.9)	7 (5.8)
Volunteering	4 (2.6)	1 (2.9)	3 (2.5)
Cancer type			
Glioma	54 (34.8)	11 (32.4)	43 (35.5)
Breast cancer	27 (17.4)	6 (17.6)	21 (17.4)
Melanoma	18 (11.6)	2 (5.9)	16 (13.2)
Lung cancer	14 (9.0)	3 (8.8)	11 (17.4)
Sarcoma	11 (7.3)	2 (5.9)	9 (7.4)
Colon cancer	8 (5.1)	2 (5.9)	6 (5.0)
Cervical cancer	8 (5.1)	4 (11.8)	4 (3.3)
Kidney cancer	2 (1.3)	0 (0.0)	2 (1.7)
Thymus cancer	2 (1.3)	0 (0.0)	2 (1.7)
Stomach	2 (1.3)	1 (2.9)	1 (0.8)
Leukemia	2 (1.3)	1 (2.9)	1 (0.8)
Neuroendocrine tumor	2 (1.3)	0 (0.0)	2 (1.7)
Other ^a^	5 (3.2)	2 (5.9)	3 (2.5)
Treatment in the past four weeks *			
No treatment	67 (43.2)	10 (29.4)	57 (47.1)
Targeted therapy	31 (20.6)	7 (20.6)	24 (19.8)
Chemotherapy	32 (20.6)	10 (29.4)	22 (18.2)
Immunotherapy	30 (19.4)	11 (32.4)	19 (15.7)
Hormonal therapy	15 (9.8)	5 (14.7)	10 (9.1)
Radiotherapy	5 (3.2)	0 (0.0)	5 (4.1)
Surgery	5 (3.2)	2 (5.9)	3 (2.5)
Previous treatment *			
Surgery	103 (66.5)	23 (67.6)	80 (66.1)
Chemotherapy	86 (55.5)	20 (58.8)	66 (54.5)
Radiotherapy	75 (48.4)	17 (50.0)	58 (47.9)
Immunotherapy	37 (23.9)	5 (14.7)	32 (26.4)
Targeted therapy	27 (17.4)	5 (14.7)	22 (18.2)
Hormonal therapy	12 (7.7)	5 (14.7)	7 (6.6)
No treatment	7 (4.5)	1 (2.9)	6 (5.0)
Hyperthermia	1 (0.6)	0 (0.0)	1 (0.8)
Additional support *			
None	16 (10.3)	2 (5.9)	14 (11.6)
Informal caregivers	73 (47.1)	22 (64.7)	51 (42.1)
General practitioner	72 (46.5)	19 (55.9)	53 (43.8)
Physiotherapist	69 (44.5)	19 (55.9)	50 (41.3)
Medical psychologist	63 (40.6)	20 (58.8)	43 (35.5)
Oncology nurse	51 (32.9)	12 (35.3)	39 (32.2)
Social worker	51 (32.9)	12 (35.3)	39 (32.2)
Peers	36 (23.2)	16 (47.1)	20 (16.5)
AYA nurse specialist	33 (21.3)	10 (29.4)	23 (19.0)
Occupational therapist	33 (21.3)	10 (29.4)	23 (19.0)
Home care	31 (20.0)	13 (38.2)	18 (14.9)
Rehabilitation physician	28 (18.1)	8 (23.5)	20 (16.5)
Alternative medicine	27 (17.4)	9 (26.5)	18 (14.9)
Occupational health physician	26 (16.8)	9 (26.5)	17 (14.0)
Fertility specialist	25 (16.1)	6 (17.6)	19 (15.7)
Dietician	8 (5.2)	2 (5.9)	6 (5.0)
Speech therapist	8 (5.2)	3 (8.8)	5 (4.1)
Palliative care team	7 (4.5)	5 (14.7)	2 (1.7)
Sexologist	5 (3.2)	1 (2.9)	4 (3.3)
Spiritual caregiver	3 (1.9)	0 (0.0)	3 (2.5)
Treatment goal according to patients *			
To extend life as long as possible (keep disease in control)	125 (80.6)	25 (73.5)	100 (82.6)
To cure	36 (23.2)	9 (26.5)	27 (22.3)
To make sure I have tried everything	31 (20.0)	11 (32.4)	20 (16.5)
To remain hopeful for me	22 (14.2)	7 (20.6)	15 (12.4)
To remain hopeful for my family	19 (12.3)	6 (17.6)	13 (10.7)
To support research	18 (11.6)	6 (17.6)	12 (9.9)
To monitor the tumor (watchful waiting)	13 (8.4)	3 (8.8)	10 (8.3)
To reduce my suffering	13 (8.4)	6 (17.6)	7 (5.8)
To manage my symptoms	4 (2.6)	3 (8.8)	1 (0.8)

Abbreviations: AYA, adolescent and young adult; UPCP, uncertain or poor cancer prognosis; * Patients were able to select multiple answers; ^a^: ovarian cancer, paraganglioma, liver cancer, pseudo myogenic hemangioendothelioma, germ cell tumor.

**Table 2:** Subscales with complete items included in the clustering analysis.

Scale	Range	Cluster 1 Median	Cluster 1 Mean (±SD)	Cluster 2 Median	Cluster 2 Mean (±SD)
GAD-7 *					
Anxiety	0–21	10.0	9.7 (6.1)	3.0	3.5 (3.6)
**PHQ-9**					
Depression	0–27	9.5	11.3 (4.9)	3.0	3.3 (3.1)
**Demoralization scale**					
Loss of meaning	0–28	11.0	12.0 (5.5)	2.0	3.3 (3.7)
Dysphoria	0–24	12.0	11.8 (3.7)	4.0	4.7 (3.5)
Disheartenment	0–32	17.5	18.4 (4.9)	6.0	6.9 (4.9)
Helplessness	0–16	8.0	8.0 (2.9)	2.0	2.8 (2.9)
Sense of failure	0–16	8.0	7.9 (2.5)	3.0	3.0 (2.6)
**EORTC QLQ-C30**					
Global quality of life	0–100	50.0	52.9 (15.2)	75.0	75.3 (18.9)
Physical functioning	0–100	76.7	71.2 (21.5)	93.3	89.9 (14.4)
Emotional functioning	0–100	41.7	43.3 (24.6)	75.0	73.4 (19.6)
Role functioning	0–100	33.3	37.3 (27.5)	83.3	73.8 (27.4)
Cognitive functioning	0–100	50.0	44.6 (27.7)	83.3	74.2 (24.5)
Social functioning	0–100	41.7	40.7 (28.8)	83.3	76.2 (24.5)
Fatigue	0–100	66.7	67.0 (23.3)	33.3	36.5 (24.5)
Nausea/vomiting	0–100	16.7	26.0 (28.8)	0.0	10.2 (20.8)
Pain	0–100	33.3	38.7 (30.9)	16.7	17.6 (23.2)
Dyspnea	0–100	16.7	23.5 (27.9)	0.0	9.1 (16.7)
Insomnia	0–100	33.3	49.0 (36.9)	0.0	25.3 (31.6)
Appetite loss	0–100	33.3	35.3 (33.8)	0.0	14.3 (25.8)
Constipation	0–100	0.0	26.5 (33.6)	0.0	15.7 (24.7)
Diarrhea	0–100	0.0	25.5 (32.9)	0.0	16.3 (26.2)
Financial difficulties	0–100	33.3	46.1 (33.8)	0.0	22.6 (28.9)
**Problems and Needs**					
ADL problems	0–7	4.5	4.8 (2.0)	1.0	2.1 (2.2)
Physical problems	0–18	10.5	10.8 (3.1)	6.0	6.6 (3.5)
Role problems	0–4	3.0	3.0 (1.0)	2.0	1.8 (1.2)
Financial problems	0–5	3.5	3.3 (1.3)	2.0	2.3 (1.3)
Social problems	0–15	9.0	8.7 (3.1)	4.0	4.8 (3.1)
Psychological problems	0–15	12.0	12.0 (2.0)	7.0	6.6 (3.2)
Spiritual problems	0–5	3.0	3.4 (0.9)	1.0	1.6 (1.2)
Autonomy problems	0–9	8.0	8.3 (0.8)	0.0	5.2 (2.9)
Information provision	0–9	5.0	5.2 (1.6)	5.0	5.2 (2.2)
**Hope**					
Hope	1–4	4.0	3.1 (1.1)	4.0	3.5 (0.9)
**PROMIS** **Meaning and Purpose**					
Meaning and purpose	20–80	39.0	38.3 (6.6)	53.4	54.5 (9.4)

* Text in bold: questionnaire names.

## Data Availability

The data presented in this study are available on request from the corresponding author due to privacy and confidentiality restrictions.

## Supplementary Materials

The following supporting information can be downloaded at: https://www.mdpi.com/article/10.3390/curroncol33070376/s1, File S1: Cluster tendency and ensemble clustering.

## Appendix A. Overview of Measures

Problems and needs. The Problems and Needs in Palliative Care Questionnaire (PNPC) is designed to assess problems and needs among palliative cancer patients. An adapted version of this questionnaire was used in this study, as the original PNPC includes 13 domains of which nine were relevant to this study: activities of daily living (ADL), physical issues, role tasks, financial problems, social problems, psychological problems, spiritual problems, autonomy, and informational needs. This adapted questionnaire includes 87 items [20]. Each item consists of two questions: whether this was a problem (yes/somewhat/no) and whether someone had a need for (more) professional support regarding this problem (yes, more/as much as now/no). For the problems and needs, an item was scored as a problem when a patient scored either ‘yes’ or ‘somewhat’. An item was scored as a need when a patient scored ‘yes, more’. Higher scores indicate increased problems or needs. Internal consistency for most dimensions is adequate (α > 0.70). Internal consistency for role tasks (α = 0.68) was lower [48].

Quality of life. For measuring quality of life, the EORTC QLQ-C30 was used, consisting of 30 items. In total, 28 items were scored on a 4-point Likert-scale (from “not at all” to “very much”) and two general quality of life items were assessed on a 7-point scale (“very poor” to “excellent”). The questionnaire includes five functioning scales (physical, emotional, role, cognitive and social) and nine items on symptoms (fatigue, nausea, pain, dyspnea, insomnia, appetite loss, constipation, diarrhea, and financial impact). Scores range from 0 and 100, with a higher score indicates better global quality of life and functioning, or a higher symptom burden [21,22].

Anxiety. The General Anxiety Disorder (GAD-7) questionnaire assesses the specific symptoms of a generalized anxiety disorder using 7 questions [23]. It focuses on the symptoms experienced by individuals in the two weeks prior to completion the questionnaire, scored on a 4-point Likert-scale (ranging from “not at all” to “almost every day”). Scores can vary from 0 to 21, with higher score indicating more severe symptoms [23]. The GAD-7 demonstrates a very good reliability (α = 0.86) and a very good divergent validity (r = 0.82). Internal consistency and convergent validity were defined as excellent [49].

Depression. Depressive symptoms were measured using the Patient Health Questionnaire (PHQ-9), which examines DSM-V criteria for a major depressive disorder. It focuses on the number of symptoms experienced by individuals in the two weeks prior to completion, using a 4-point Likert-scale (from “not at all” to “nearly every day”) [24]. Total scores range from 0 to 27, with scores from 5 to 9 indicating mild, 10 to 14 moderate, and 15 to 27 severe depressive symptoms. Internal consistency of the PHQ-9 was classified as good (α = 0.86) and had strong correlations with other questionnaires measuring the same construct (r between 0.63 and 0.73) [50].

Demoralization. The Demoralization Scale (DS) is a 24-item questionnaire to assess existential distress in advanced cancer patients. Items are rated from 0 to 4 (from “never” to “all the time”). The subscales include a loss of meaning, dysphoria, disheartenment, helplessness, and sense of failure, with higher scores indicating an increased burden among these domains. Cronbach’s alpha of all subscales is acceptable, ranging from 0.71 to 0.89. Convergent and divergent validity are also acceptable [25].

Meaning and purpose. The PROMIS Meaning and Purpose consists of 8 items that measure how much an individual feels like their life matters or is meaningful to them. Each item is rated on a 5-point Likert-scale (ranging from “strongly disagree” to “strongly agree”). The raw score of this questionnaire is converted to a T-score, compared to a reference population of a healthy general population. Higher T-scores indicate a stronger feeling of meaning and purpose in life. This questionnaire demonstrates excellent reliability (α = 0.92), and strong model fit [26].

Hope. Hope was measured using a self-developed item based on our previous qualitative work [27], and advice from our patient panel including AYAs with an UPCP. The item, “I believe that something will happen that will cure me”, was scored on a 4-point Likert scale (from “not at all” to “very much”). This item assesses the hope one has for their future and the feeling of being “an exception”. Higher score indicated more hope.

Life urgency. Life-urgency was also measured using a self-developed item based on our previous qualitative work [27] and feedback from our patient panel: “I feel the urge to do as many things as possible in a short amount of time because of my uncertain or poor prognosis”. Responses are scored from 1 to 4 (“not at all” to “very much”). This item measured the sense of urgency associated with a shortened life span, with higher score indicating greater life urgency.

Goal adjustment. The Goal Adjustment Scale (GAS) is a 10-item questionnaire on one’s ability to disengage and reengage with goals when they are required to stop working on them [28]. This scale can be divided into two subscales: goal disengagement and goal reengagement. Scores for each item range from 1 to 5 (ranging from “almost never true” to “almost always true”). The psychometric properties of this scale show good reliability and validity [51].

Illness uncertainty. The Mishel Uncertainty in Illness Scale (MUIS-A) questionnaire was designed to measure uncertainty related to illness. It consists of 24 items addressing one’s symptoms, disease trajectory, social support and their future. Items are scored on a scale from 1 to 5 (ranging from “strongly disagree” to “strongly agree”), with higher scores indicating one experiences more uncertainty. Furthermore, it can be divided into two subscales: lack of clarity and unpredictability. This questionnaire demonstrated good reliability and convergent validity [29].

Self-efficacy. Self-efficacy was assessed using the Self-Efficacy for Managing Chronic Disease, which includes 6 items addressing fatigue, pain, emotional distress, health problems, tasks to manage health problems and other medical interventions, and how an individual manages them [30]. Respondent rate their confidence to manage these challenges on a scale from 0 to 10 (ranging from “not at all confident” to “totally confident”). A score was derived using the mean of all six items. This scale is applicable to various diseases, including cancer, and was proven to have good internal consistent reliability [52].

Coping. Coping strategies were measured using the BRIEF-COPE questionnaire, designed to assess coping in patients with different diseases. It covers 14 coping domains, with two items per domain, totaling 28 items. The domains consist of self-distraction, denial, substance use, behavioral disengagement, emotional support, venting, humor, acceptance, self-blame, religion, active coping, use of instrumental support, positive reframing and planning. Higher scores indicate increased usage of a certain coping strategy. Cronbach’s alpha for the scales range from acceptable to high (0.50 to 0.90) [31].

Resilience. Resilience was examined using the 10-item Connor Davidson Resilience Scale (CD-RISC-10), which measures an individual’s ability to persevere in times of adversity. Items are rated on a scale from 0 to 4 (ranging from “not true at all” to “true nearly all the time”), with higher scores indicating more resilience. The scale has a Cronbach’s alpha of 0.86, indicating good internal consistency, and also demonstrates good convergent validity [32].

Social support. Social support was assessed using an adapted version of the Duke-UNC Functional Social Support Questionnaire (FSSQ). This modified version includes five items, with three general items combined into one focusing on discussing the disease and disease-related problems with others. Responses are scored on a scale from 1 to 5 (ranging from “much less than I would like” to “As much as I would like”), with higher scores indicating greater social support [33].

## Appendix B. External Items, Removed Due to Missing Items

**Table A1:** External factors among AYAs with a UPCP.

Scale	Range	Cluster 1 Median	Cluster 1 Mean (±SD)	Cluster 2 Median	Cluster 2 Mean (±SD)
**Goal adjustment scale ***					
Disengagement	4–20	12.0	11.8 (1.6)	12.0	11.7 (1.7)
Reengagement	6–30	27.0	25.6 (3.8)	29.0	27.5 (2.8)
**Illness uncertainty scale**					
Total score	0–96	71.5	71.6 (10.8)	58.0	58.0 (11.8)
Lack of clarity	0–44	30.0	29.0 (5.9)	24.0	24.1 (5.5)
Unpredictability	0–76	60.0	59.5 (8.6)	48.0	47.6 (9.9)
**Self-efficacy**					
Self-efficacy	1–10	4.0	3.7 (2.0)	6.5	6.4 (1.9)
**BRIEF-COPE**					
Problem-focused coping	6–24	23.0	21.4 (4.7)	23.0	22.9 (4.5)
Active coping	2–8	6.0	5.8 (1.6)	6.0	6.1 (1.6)
Planning	2–8	6.0	5.5 (1.6)	6.0	5.6 (1.7)
Informational support	2–8	6.0	5.1 (1.5)	6.0	5.7 (1.7)
Emotion-focused coping	12–48	29.3	29.3 (5.3)	29.0	29.4 (4.3)
Emotional support	2–8	6.0	5.9 (1.7)	7.0	6.8 (1.4)
Positive reframing	2–8	5.0	5.0 (1.8)	6.0	5.5 (1.7)
Acceptance	2–8	6.0	5.6 (1.7)	7.0	6.5 (1.4)
Religion	2–8	3.0	3.2 (1.6)	3.0	3.7 (2.0)
Humor	2–8	4.0	4.2 (1.2)	4.0	4.2 (1.5)
Venting	2–8	6.0	5.3 (1.4)	5.0	4.6 (1.5)
Avoidant coping	10–40	16.0	16.3 (4.1)	13.0	13.9 (3.3)
Self-distraction	2–8	5.0	5.8 (1.5)	6.0	5.7 (1.5)
Denial	2–8	4.0	3.6 (1.6)	2.0	2.8 (1.2)
Behavioral disengagement	2–8	3.0	3.6 (1.5)	2.0	2.6 (1.2)
Substance use	2–8	2.0	3.2 (1.7)	2.0	2.8 (1.5)
Self-blame	2–8	5.0	5.2 (1.9)	3.0	3.5 (1.5)
**CD-RISC-10**					
Resilience	0–40	22.0	22.2 (6.2)	30.0	30.7 (5.6)
**FSSQ**					
Social support	1–5	4.2	3.8 (1.1)	4.8	4.6 (0.5)
**Problems and Needs**					
ADL needs	0–7	1.0	1.7 (1.9)	0.0	0.4 (1.1)
Physical needs	0–18	5.0	4.6 (2.9)	1.0	1.7 (2.5)
Role needs	0–4	3.0	1.9 (1.3)	0.0	0.7 (1.1)
Financial needs	0–5	2.2	2.2 (1.5)	1.0	1.2 (1.4)
Social needs	0–15	6.1	6.1 (4.2)	1.0	2.1 (3.0)
Psychological needs	0–15	7.7	7.7 (4.6)	1.0	2.1 (2.8)
Spiritual needs	0–5	2.0	2.1 (1.4)	0.0	0.5 (1.0)
Autonomy needs	0–9	5.0	4.7 (3.1)	0.0	1.6 (2.3)
Informational needs	0–9	5.0	5.2 (2.3)	3.0	2.5 (2.5)
**Life-urgency**					
Life-urgency	1–4	3.0	2.6 (1.1)	2.0	2.0 (1.0)

* Bold: names of questionnaires.
