# Supplementary material for: Coping with an Uncertain or Poor Cancer Prognosis as an Adolescent or Young Adult: A Cross-Sectional Cluster Analysis
Source: Curr Oncol. 2026 Jun 23;33(7):376. doi: 10.3390/curroncol33070376 (PMC13409294; doi:10.3390/curroncol33070376)
Supplement: Supplementary file 1 [file curroncol-33-00376-s001.zip › curroncol-4319101-SI.pdf]

Cluster Tendency and ensemble clustering

Leyla Azarang l.azarang@nki.nl

2025-10-27

Table of Contents

CORD-AYA Data 1
Is data from CORD-AYA study suitable for clustering analysis? 4
Data set with complete variables 4
Data set with at most 5% missings 4
Ensembel clustering 5
Evaluation 5
Optimal number of clusters 6
Optimal algorithms 6
Are two clusters really there? 9
CORD-AYA clusters 9
References 11
Technical information 11

CORD-AYA Data

In this study, 155 adolescents and young adults (AYAs) with an uncertain or poor cancer prognosis (UPCP) were included.

I explored two sub-datasets from the original study:

- CORD_AYA_notext_withnomiss: that is the data with complete items, containing 155 patients and 220 items.

It includes the following items/variables: ID, Date_birth, Sex, CA_partner, Living, Edu, Student, Fulltime, Parttime, Unemploy, Homemaker, Sickleave, Disabled, CA_work, Relig, Curr_surg, Curr_chemo, Curr_radio, Curr_immuno, Curr_horm, Curr_target, Curr_other, Prev_surg, Prev_chemo, Prev_radio, Prev_immuno, Prev_horm, Prev_targ, Prev_treat, Aimcure, Aimprolong, Hopeme, Aimhopefam, Aimtry, Aimsuffer, Aimresearch, Aimtreat, Tumortype, Supno, Supnursespec, Suponconurse, Supsw, Supphysther, Supoccupther, Supmp, Suplogo, Suprehab, Supfert, Supoccupphys, Supgp, Suphome, Supsex, Suppal, Suppeers, Supinfcg, Supalt, Support, ADLprob, Walkprob, Mealprob, Shopprob, Transprob, Houselightprob, Heavyhouseprob, Painprob, Concentrationprob, Fatigueprob, Sleepprob, Nauseaprob, Constipationprob, Incontinenceprob, Mouthprob, Appetiteprob, Dyspneaprob, Coughprob,

Itchprob, Hairlossprob, Visionprob, Sexprob, Numbprob, Edemaprob, Sweatprob, Filldayprob, Relaxprob, Employprob, Childprob, Costsprob, Incomeprob, Arrangeprob, Formsprob, Financialtalkprob, Relationprob, Talkpartnerprob, Talkchildprob, Talkothersprob, Burdenprob, Receptiveprob, Opinionprob, Supprob, Confidantprob, Practicalprob, Concernedprob, Dramatizeprob, Severityprob, Lonelyprob, Forsakenprob, Depressedprob, Pleasureprob, Treatmentprob, Metastasizeprob, Aloneprob, Deathprob, Unpredictprob, Emotionsprob, Guiltprob, Shameprob, Controlprob, Appearanceprob, Positiveprob, Overwhelmedprob, Engageprob, Availableprob, Godprob, Meaningdeathprob, Acceptprob, Usualprob, Socialprob, Handoverprob, Dependentprob, Frustrationprob, Bodycontrolprob, Lifecontrolprob, Askhelpprob, Decisionsprob, Aidsprob, Placesprob, Causeprob, Symptomsprob, Alternativeprob, Euthanasiaprob, Nourishprob, Infosexprob, QL_Activities, QL_Longwalk, QL_Shortwalk, QL_Stayseated, QL_ADL, QL_Work, QL_Hobby, QL_Dyspnoea, QL_Pain, QL_Resting, QL_Sleeping, QL_Weak, QL_Appetite, QL_Nausea, QL_Vomit, QL_constipate, QL_diarrhea, QL_tired, QL_dailypain, QL_concentrate, QL_tense, QL_worry, QL_irritate, QL_depress, QL_remember, QL_familylife, QL_sociallife, QL_financial, QL_overallhealth, QL_overallqol, Anx_nervous, Anx_controlworry, Anx_worrying, Anx_relaxing, Anx_restless, Anx_annoyed, Anx_afraid, Dep_anhedonia, Dep_down, Dep_sleep, Dep_tired, Dep_appetite, Dep_fail, Dep_concentrate, Dep_slow, Dep_suicide, Dem_valuable, Dem_pointless, Dem_purpose, Dem_role, Dem_controlemo, Dem_spirit, Dem_helpother, Dem_helpless, Dem_hopeless, Dem_guilt, Dem_irritable, Dem_coping, Dem_regrets, Dem_worthlife, Dem_feelhurt, Dem_anger, Dem_proud, Dem_distress, Dem_worthy, Dem_suicidal, Dem_sad, Dem_discourage, Dem_alone, Dem_trapped, Mean_meaning, Mean_fulfillment, Mean_purpose, Mean_meaningful, Mean_goals, Mean_significance, Mean_worthwhlie, and Hope.

- CORD_AYA_notext_less5miss: that is the data with items that have less than 5% missing information. This dataset contains 155 patients and 397 items. However, after removing the missing information, the number of patients decreases to 143. I do not impute the missing information for this data, instead I use a deletion strategy (remove the patients who have missing in at least one item).

It includes the following items/variables: ID, Date_birth, Sex, CA_partner, Living, Edu, Student, Fulltime, Parttime, Unemploy, Homemaker, Sickleave, Disabled, CA_work, Relig, Age_diag, Age_treat, Curr_surg, Curr_chemo, Curr_radio, Curr_immuno, Curr_horm, Curr_target, Curr_other, Prev_surg, Prev_chemo, Prev_radio, Prev_immuno, Prev_horm, Prev_targ, Prev_treat, Aimcure, Aimprolong, Hopeme, Aimhopefam, Aimtry, Aimsuffer, Aimresearch, Aimtreat, Tumortype, Supno, Supnursespec, Suponconurse, Supsw, Supphysther, Supoccupther, Supmp, Suplogo, Suprehab, Supfert, Supoccupphys, Supgp, Suphome, Supsex, Suppal, Suppeers, Supinfcg, Supalt, Support, ADLprob, ADLneed, Walkprob, Walkneed, Mealprob, Mealneed, Shopprob, Shopneed, Transprob, Transneed, Houselightprob, Houselightneed, Heavyhouseprob, Heavyhouseneed, Painprob, Painneed, Concentrationprob, Concentrationneed, Fatigueprob, Fatigueneed, Sleepprob, Sleepneed, Nauseaprob, Nauseaneed, Constipationprob, Constipationneed, Incontinenceprob, Incontinenceneed, Mouthprob, Mouthneed, Appetiteprob, Appetiteneed, Dyspneaprob, Dyspneaneed, Coughprob, Coughneed, Itchprob, Itchneed, Hairlossprob, Hairlossneed, Visionprob, Visionneed, Sexprob, Sexneed, Numbprob, Numbneed, Edemaprob, Edemaneed, Sweatprob, Sweatneed, Filldayprob, Filldayneed, Relaxprob, Relaxneed, Employprob, Employneed, Childprob, Childneed, Costsprob, Costsneed, Incomeprob, Incomeneed, Arrangeprob, Arrangeneed, Formsprob, Formneed, Financialtalkprob,

Financialtalkneed, Relationprob, Relationneed, Talkpartnerprob, Talkpartnerneed, Talkchildprob, Talkchildneed, Talkothersprob, Talkotherneed, Burdenprob, Burdeneed, Receptiveprob, Receptiveneed, Opinionprob, Opinionneed, Supprob, Supneed, Confidantprob, Confidantneed, Practicalprob, Practicalneed, Concernedprob, Concernedneed, Dramatizeprob, Dramatizeneed, Severityprob, Severityneed, Lonelyprob, Lonelyneed, Forsakenprob, Forsakenneed, Depressedprob, Depressedneed, Pleasureprob, Pleasureneed, Sufferprob, Sufferneed, Treatmentprob, Treatmentneed, Metastasizeprob, Metastasizeneed, Aloneprob, Aloneneed, Deathprob, Deathneed, Unpredictprob, Unpredictneed, Emotionsprob, Emotionsneed, Guiltprob, Guiltneed, Shameprob, Shameneed, Controlprob, Controlneed, Appearanceprob, Appearanceneed, Positiveprob, Positiveneed, Overwhelmedprob, Overwhelmedneed, Engageprob, Engageneed, Availableprob, Availableneed, Godprob, Godneed, Meaningdeathprob, Meaningdeathneed, Acceptprob, Acceptneed, Usualprob, Usualneed, Socialprob, Socialneed, Handoverprob, Handoverneed, Dependentprob, Dependentneed, Frustrationprob, Frustrationneed, Bodycontrolprob, Bodycontrolneed, Lifecontrolprob, Lifecontrolneed, Askhelpprob, Askhelpneed, Decisionsprob, Decisionneed, Aidsprob, Aidsneed, Placesprob, Placesneed, Causeprob, Causeneed, Optionsprob, Optionsneed, Symptomsprob, Symptomsneed, Alternativeprob, Alternativeneed, Euthanasiaprob, Euthanasianeed, Nourishprob, Nourishneed, Infosexprob, Infosexneed, QL_Activities, QL_Longwalk, QL_Shortwalk, QL_Stayseated, QL_ADL, QL_Work, QL_Hobby, QL_Dyspnoea, QL_Pain, QL_Resting, QL_Sleeping, QL_Weak, QL_Appetite, QL_Nausea, QL_Vomit, QL_constipate, QL_diarrhea, QL_tired, QL_dailypain, QL_concentrate, QL_tense, QL_worry, QL_irritate, QL_depress, QL_remember, QL_familylife, QL_sociallife, QL_financial, QL_overallhealth, QL_overallqol, Anx_nervous, Anx_controlworry, Anx_worrying, Anx_relaxing, Anx_restless, Anx_annoyed, Anx_afraid, Dep_anhedonia, Dep_down, Dep_sleep, Dep_tired, Dep_appetite, Dep_fail, Dep_concentrate, Dep_slow, Dep_suicide, Dem_valuable, Dem_pointless, Dem_purpose, Dem_role, Dem_controlemo, Dem_spirit, Dem_helpother, Dem_helpless, Dem_hopeless, Dem_guilt, Dem_irritable, Dem_coping, Dem_regrets, Dem_worthlife, Dem_feelhurt, Dem_anger, Dem_proud, Dem_distress, Dem_worthy, Dem_suicidal, Dem_sad, Dem_discourage, Dem_alone, Dem_trapped, Mean_meaning, Mean_direction, Mean_fulfillment, Mean_purpose, Mean_meaningful, Mean_goals, Mean_significance, Mean_worthwhlie, Hope, Urg, Goal_effort, Goal_othergoal, Goal_commitment, Goal_newgoal, Goal_makegoal, Goal_stopgoal, Goal_meaning, Goal_letgo, Goal_focus, Goal_effortother, Insec_happening, Insec_questions, Insec_illness, Insec_severepain, Insec_unclearinfo, Insec_goaltreat, Insec_changesymp, Insec_understandinfo, Insec_diffmeaning, Insec_complextreat, Insec_effecttreat, Insec_unpredict, Insec_changingcourse, Insec_opinions, Insec_uncleardirect, Insec_resultsinconsistent, Insec_effectiveness, Insec_makeplans, Insec_othersolut, Insec_succestreat, Insec_diagnosis, Insec_severity, Insec_language, Insec_sideeffect, Effic_fatigue, Effic_pain, Effic_distress, Effic_symptoms, Effic_visitdr, Effic_medication, Cope_work, Cope_engage, Cope_notreal, Cope_substance, Cope_emosup, Cope_gaveup, Cope_action, Cope_refuse, Cope_speaking, Cope_advice, Cope_alcohol, Cope_reframe, Cope_criticize, Cope_strategy, Cope_someone, Cope_giveup, Cope_findgood, Cope_joke, Cope_distract, Cope_accept, Cope_negative, Cope_religion, Cope_helpother, Cope_learnlive, Cope_takestep, Cope_blame, Cope_pray, Cope_makefun, Res_adapt, Res_deal, Res_humorous, Res_cope, Res_bounceback, Res_achieve, Res_focus, Res_discourage, Res_strong, Res_handleemo, Soc_care, Soc_love, Soc_talking, Soc_dothings, Soc_advice, and Soc_sick.

- Only one patient has all the information completed.

Is the data from the CORD-AYA study suitable for clustering analysis?

In this section, I use Hopkins statistic (Lawson and Jurs, 1990) to assess the clustering tendency of the datasets.

Here, the null and alternative hypothesis are as follows:

- Null hypothesis: the data set D is uniformly distributed (i.e., no meaningful clusters)
- Alternative hypothesis: the data set D is not uniformly distributed (i.e., contains meaningful clusters).

I can conduct the Hopkins Statistic test, using 0.5 as the threshold to reject the alternative hypothesis. That is, if $H < 0.5$, it is unlikely that our data set has statistically significant clusters. If the value of Hopkins statistic is larger than 70, I can reject the null hypothesis and conclude that the dataset is a significantly clusterable dataset with a significant level equal to 0.01.

Data set with complete variables

```
CORD_AYA_notext_withnomiss <- CORD_AYA_notext_withnomiss[,-1] %>%
  map_df(as.numeric) %>%
  mutate(ID=CORD_AYA_notext_withnomiss$ID ) %>%
  relocate(ID)

gradient.color <- list(low = "steelblue",  high = "white")
clust_tend_comp <- CORD_AYA_notext_withnomiss[,-1]  %>%
scale() %>%
get_clust_tendency(n = 15, gradient = gradient.color, seed = 432)
```

Since the Hopkins statistic is 0.59, I cannot conclude that our dataset exhibits statistically significant clustering. However, I also cannot confidently say that it lacks cluster tendency, as the value does not fall below the typical threshold for randomness (0.5), nor does it exceed the 0.7 threshold commonly used to indicate strong clusterability.

Data set with at most 5% missing

```
CORD_AYA_notext_less5miss <- CORD_AYA_notext_less5miss %>%
  drop_na()

CORD_AYA_notext_less5miss <-CORD_AYA_notext_less5miss[,-1] %>%
map_df(as.factor) %>%
  mutate(ID=CORD_AYA_notext_less5miss$ID ) %>%
relocate(ID)

df <- CORD_AYA_notext_less5miss[,-1] %>%
map_df(as.numeric) %>%
  mutate(ID=CORD_AYA_notext_less5miss$ID ) %>%
relocate(ID)
```

```
gradient.color <- list(low = "red",  high = "white")
clust_tende_less5miss <- df[, -1] %>%
scale() %>%
get_clust_tendency(n = 14, gradient = gradient.color, seed = 432)
```

Hopkins statistics for this data set is 0.58. This is slightly less than that of the dataset with complete variables.

Now, from the two candidate datasets, I can only cluster one. I choose the one with complete items because of having slightly higher Hopkin's statistics, including all patients and exhibiting lower multicollinearity.

Ensemble clustering

As I discussed in the previous section, there is no strong evidence that the data with complete items is significantly clusterable. However, in the context of our data, a weak Hopkins score may still be acceptable if very small number of clusters (groupings) are meaningful. That can be two/three paths in the case of CORD-AYA data.

In this section, I perform a cluster analysis by applying an ensemble clustering framework using diceR package. This framework enables the user to choose an ensemble from diverse set of algorithms for clustering. Then, the pooled result from the chosen algorithms will conclude the final clusters.

I applied the ensemble clustering of the following algorithms: K-Means, Partition Around Medoids, Hierarchical agglomerative, hierarchical divisive, and a neural network model (Self-Organizing Map (SOM) with Hierarchical Clustering) clustering. The distance functions for Partition around Medoids, and hierarchical models are "manhattan" and "euclidean".

Here, I consider 3 different number of clusters (k=2,3,4). Also, I use 80% of the data for sub-sampling, then repeat the sub-sampling 5 times and apply the algorithms to each sub-sample. That is, every algorithm is applied to 5 subsets of the data, each consisting of 80% of the original observations. As a result of sub-sampling, not every observation is included in each clustering: the data is "completed" using k-nearest neighbor and majority voting.

The method I use ensures that the data has been examined from several perspectives and through diverse approaches.

I use diceR package to apply this method to our data.

```
CC_CORD_AYA_notext_withnomiss <- consensus_cluster( CORD_AYA_notext_withnomiss[,-1], nk
= 2:4, p.item = 0.8, reps = 5,algorithms = c("km","pam","hc", "diana",  "som")
,   hc.method = "ward.D2", distance = c("manhattan", "euclidean"),  scale = TRUE)
```

Evaluation

```
int_val_CORD_AYA_notext_withnomiss <- consensus_evaluate(CORD_AYA_notext_withnomiss[,-1],
CC_CORD_AYA_notext_withnomiss, plot = FALSE)
```

```
int_val_CORD_AYA_notext_withnomiss2 <- consensus_evaluate(CORD_AYA_notext_withnomiss[,-1
], CC_CORD_AYA_notext_withnomiss, trim = TRUE, n=3, plot = FALSE)
str(int_val_CORD_AYA_notext_withnomiss2 , max.level = 2)
```

```
## List of 5
##  $ k       : int 2
##  $ pac     :'data.frame':    3 obs. of  9 variables:
##   ..$ k              : chr [1:3] "2" "3" "4"
##   ..$ PAM_Manhattan  : num [1:3] 0.0916 0.2411 0.2214
##   ..$ PAM_Euclidean  : num [1:3] 0.413 0.183 0.256
##   ..$ HC_Manhattan   : num [1:3] 0.228 0.328 0.321
##   ..$ HC_Euclidean   : num [1:3] 0.493 0.348 0.319
##   ..$ DIANA_Manhattan: num [1:3] 0.0196 0.1531 0.2787
##   ..$ DIANA_Euclidean: num [1:3] 0.0691 0.3172 0.2014
##   ..$ KM             : num [1:3] 0.2561 0.0672 0.3045
##   ..$ SOM            : num [1:3] 0.4 0.358 0.376
##  $ ii      :List of 3
##   ..$ 2:'data.frame':    8 obs. of  16 variables:
##   ..$ 3:'data.frame':    8 obs. of  16 variables:
##   ..$ 4:'data.frame':    8 obs. of  16 variables:
##  $ ei      : NULL
##  $ trim.obj:List of 5
##   ..$ alg.keep   : chr [1:3] "DIANA_Manhattan" "DIANA_Euclidean" "HC_Manhattan"
##   ..$ alg.remove : chr [1:5] "PAM_Manhattan" "PAM_Euclidean" "HC_Euclidean" "KM" ...
##   ..$ rank.matrix:List of 1
##   ..$ top.list   :List of 1
##   ..$ E.new      :List of 1
```

Optimal number of clusters

In order to assess the optimal number of clusters I use the Proportion of Ambiguous Clusters (PCA). This is a robust way to assess clustering performance.

```
rowMeans(int_val_CORD_AYA_notext_withnomiss2$pac[,-1])

## [1] 0.2462191 0.2494344 0.2847507
```

The smallest PAC corresponds to k =2.

Optimal algorithms

Before combining the result of each clustering algorithm to conclude the cluster of each sample, I will examine which clustering algorithms are the most appropriate for our data.

To this end, I use clustering validation indices to evaluate each clustering algorithm. I want the average distance within each cluster to be as small as possible, and the average distance between clusters to be as large as possible. There are several clustering validation indices such as: “calinski_harabasz”, “dunn”, “pbm”, “tau”, “gamma”, “c_index”, “mcclain_rao”, “sd_dis”, “ray_turi”, “g_plus”, “Compactness”and “Connectivity”.

- Indices that should be maximized are: “calinski_harabasz”, “dunn”, “pbm”, “tau”, and “gamma”.
- The indices that should be minimized are: “c_index”, “mcclain_rao”, “sd_dis”, “ray_turi”, “g_plus”, “Compactness”and “Connectivity”.

Some algorithms perform too poorly to deserve membership in the cluster ensemble. In the output above I see that some algorithm should be removed. This suggestion is based on the total rank, that is first the relative ranks of each algorithm across all internal indices are considered, then the sum of the ranks is computed. All algorithms below a certain quantile for the sum rank are trimmed (removed). By default this quantile is 75%.

```
int_val_CORD_AYA_notext_withnomiss$ii$"2"

##                          Algorithms calinski_harabasz      dunn      pbm
## PAM_Manhattan       PAM_Manhattan          23.42951 0.4102912 29.29658
## PAM_Euclidean       PAM_Euclidean          22.95122 0.3460461 24.84732
## HC_Manhattan        HC_Manhattan           23.37331 0.4156900 32.10944
## HC_Euclidean        HC_Euclidean           25.15513 0.1882938 27.43042
## DIANA_Manhattan DIANA_Manhattan          21.35520 0.4314813 37.41569
## DIANA_Euclidean DIANA_Euclidean          24.05776 0.4044942 37.40706
## KM                               KM          27.64172 0.3594464 31.69382
## SOM                             SOM          25.86771 0.3000000 31.17122
                      tau       gamma   c_index davies_bouldin mcclain_rao
## PAM_Manhattan    2.005367e-06  -6.2500000 0.2288473       2.369836   0.8344834
## PAM_Euclidean   -2.541830e-06   1.3913044 0.3268312       2.495617   0.8937298
## HC_Manhattan    -1.505283e-06  12.3333330 0.2223171       2.259289   0.8281594
## HC_Euclidean     2.105211e-06  -0.8983051 0.2444174       2.433566   0.8466597
## DIANA_Manhattan  1.206400e-06   0.6511628 0.1983895       2.101251   0.8080790
## DIANA_Euclidean  1.253479e-06  -2.7272727 0.1803970       2.109745   0.8027132
## KM               3.581775e-07   0.4285714 0.2014409       2.280786   0.8221547
## SOM             -1.798375e-06 -11.2500000 0.1815023       2.324320   0.8104264
##                     sd_dis ray_turi        g_plus silhouette Compactness
## PAM_Manhattan    0.1980996 1.396014  9.829232e-07  0.1271765    16.68602
## PAM_Euclidean    0.2153918 1.654855 -1.544594e-06  0.1171620    16.65238
## HC_Manhattan     0.1891767 1.273493 -1.319925e-06  0.1372677    16.69479
## HC_Euclidean     0.2054565 1.487084  2.246681e-07  0.1226019    16.58526
## DIANA_Manhattan 0.1739871 1.089665  2.106264e-07  0.1531533    16.80604
## DIANA_Euclidean 0.1752787 1.089023 -5.757121e-07  0.1532368    16.68638
## KM              0.1924616 1.286957 -1.628844e-06  0.1364917    16.49783
## SOM             0.1929105 1.305791 -1.109299e-06  0.1337846    16.57477
##                 Connectivity
## PAM_Manhattan       48.98135
## PAM_Euclidean       78.48254
## HC_Manhattan        36.05000
## HC_Euclidean        68.43968
## DIANA_Manhattan     35.61667
## DIANA_Euclidean     39.80040
## KM                  62.94563
## SOM                 66.11310
```

```
ind_k_2 <- int_val_CORD_AYA_notext_withnomiss$ii$"2"

ind_k_2 <- ind_k_2 %>%
   select(Algorithms,  dunn, pbm,  silhouette,   davies_bouldin,    sd_dis, ray_turi,  Compactness, Connect
ivity) %>%
     mutate(across(where(is.numeric), ~ scale(.)[, 1]))

ind_k_2_mat <- as.matrix(ind_k_2[,-1])
```

For the number of cluster equal to two (k= 2), I can see some of the indices for internal validity of the clustering algorithms. In the following heatmap I can see the internal validation indices for each algorithm. The bluer, the smaller and the redder, the bigger.

```
heatmap.2(ind_k_2_mat, scale= "none" , col = bluered(100), ColSideColors = c(rep("red", 3), rep("blue
",5)), cexRow=0.8, cexCol=0.8,  Rowv = NA, Colv = NA,  trace = "none", density.info = "none", main =
"Internal validity indices", cex.main = 0.1)
```

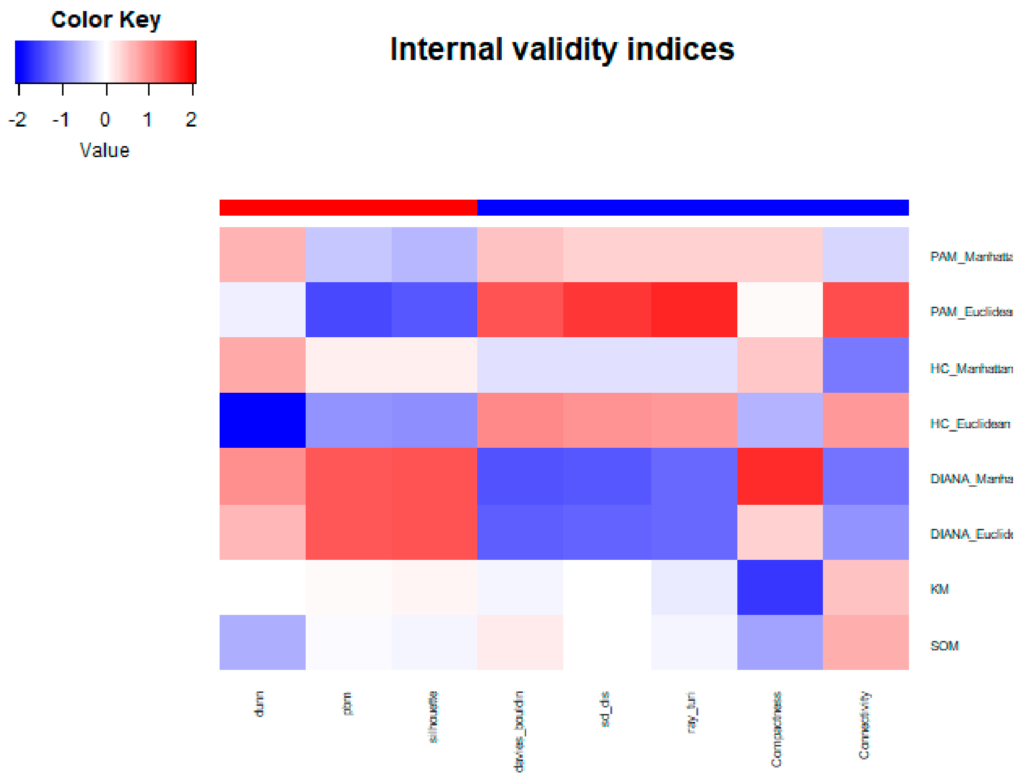

Algorithms suggested to keep are “DIANA_Manhattan” “DIANA_Euclidean” “HC_Manhattan”.

Hence, I choose these three optimal clusters (“DIANA_Manhattan”, “DIANA_Euclidean” “HC_Manhattan”)) and use Majority Voting method for pooling the results of them.

```
ccomb_class_CORD_AYA_notext_withnomiss <- consensus_combine(CC_CORD_AYA_notext_withn
omiss, element = "class")

cc_2_CORD_AYA_notext_withnomiss <- majority_voting(ccomb_class_CORD_AYA_notext_withno
miss[[1]][, c("DIANA_Manhattan", "DIANA_Euclidean", "HC_Manhattan")])
```

Are two clusters really there?

Now, I use SigClust test to examine whether there are 2 statistically distinct clusters (k = 2) versus no clusters (k = 1).

Not to mention that the null hypothesis is: “The data come from a single multivariate Gaussian (normal) distribution — i.e., there is no real cluster structure in the data.”

```
sig_clust_CORD_AYA_notext_withnomiss <- sigclust(CORD_AYA_notext_withnomiss[,-1], nsim = 10
00, labflag = 1, label = cc_2_CORD_AYA_notext_withnomiss )

sig_clust_CORD_AYA_notext_withnomiss@pvalnorm

## [1] 6.677976e-66
```

According to the above p-value, I can reject the null hypothesis that there is no cluster.

CORD-AYA clusters

```
CORD_AYA_notext_withnomiss_clust <- CORD_AYA_notext_withnomiss %>%
  mutate(label = factor(cc_2_CORD_AYA_notext_withnomiss) ) %>%
  relocate(label, .after = ID)

clusters_ensemble_model <- CORD_AYA_notext_withnomiss_clust[, 1:2]

need_data <-CORD_AYA %>%
  select(ID, Sleepneed, Relaxneed, Pleasureneed, Relationneed, Supneed) %>%
  mutate(across(ends_with("need"), as.factor)) %>%
  mutate(Supneed = factor(ifelse(Supneed == 1, "more", ifelse(Supneed ==3, "no", "as much as now")))
)

need_and_cluster_data <- need_data %>% left_join( clusters_ensemble_model, by = "ID")

tb <- table(need_and_cluster_data$Supneed, need_and_cluster_data$label , useNA = "ifany")

ft <- fisher.test(tb)
ft

##
##  Fisher's Exact Test for Count Data
##
## data:  tb
## p-value = 0.0004304
## alternative hypothesis: two.sided
```

There is a statistically significant association between the need for socials support and the pathways. The majority of patients who do not require additional attention regarding social support tend to be on the better path, and the number patients who require more social support is equal for both the poorer and better pathway.

```
p_val_fish <- round(ft$p, 5)
```

```r
unique(need_and_cluster_data$Supneed)

## [1] more          as much as now no            <NA>
## Levels: as much as now more no

need_and_cluster_data <- need_and_cluster_data %>%
   mutate(Supneed = factor(Supneed, c("no", "as much as now", "more")))

gp <-need_and_cluster_data %>%
   mutate(cluster =label) %>%
   ggplot( aes(x = Supneed)) +
   geom_bar(aes(fill= cluster), width = 0.7 ) +
  theme_minimal(base_size = 12) +  # clean theme with larger base font
  labs(
   x = "social support need",
   y = "number of patients"
  ) +
    scale_fill_brewer(palette="Paired") +
   annotate("text", x = 3, y = 50, label = paste("p-value =",p_val_fish), color = "red", size = 5, fontface =
"bold")

gp
```

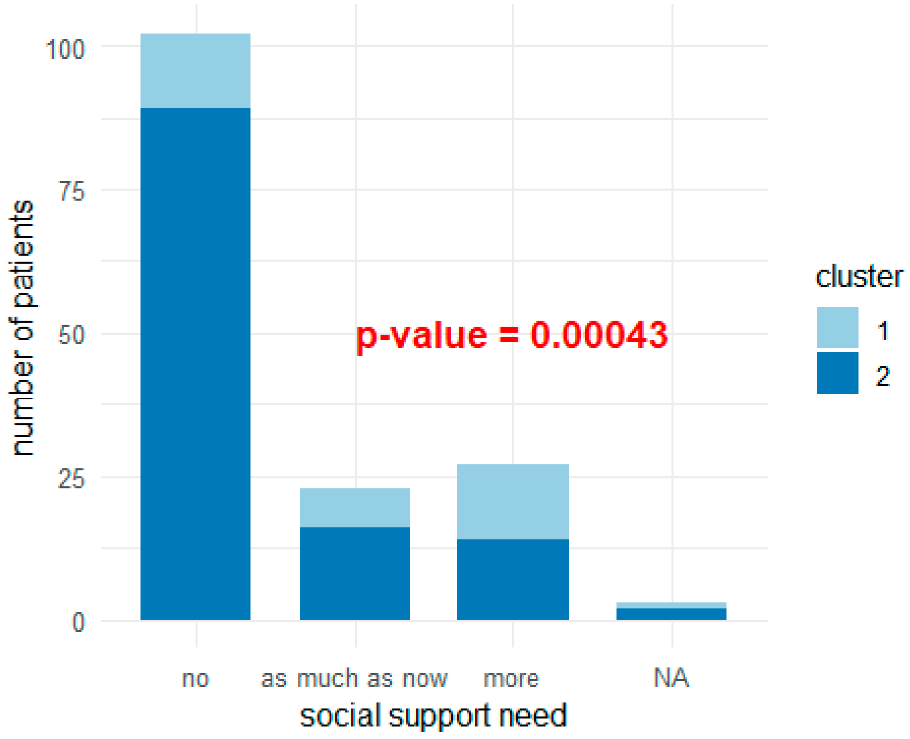

References

- Lawson, R.G. and Jurs, P.C. (1990) New Index for Clustering Tendency and Its Application to Chemical Problems. Journal of Chemical Information and Computer Sciences, 30, 36-41. https://doi.org/10.1021/ci00065a010
- Chiu DS, Talhouk A. diceR: an R package for class discovery using an ensemble driven approach. BMC Bioinformatics. 2018 Jan 15;19(1):11. doi: 10.1186/s12859-017-1996-y. PMID: 29334888; PMCID: PMC5769335.

Technical information

```
sessionInfo()
```

```
## R version 4.5.1 (2025-06-13 ucrt)
## Platform: x86_64-w64-mingw32/x64
## Running under: Windows 11 x64 (build 22631)
##
## Matrix products: default
##   LAPACK version 3.12.1
##
## locale:
## [1] LC_COLLATE=English_Netherlands.utf8  LC_CTYPE=English_Netherlands.utf8
## [3] LC_MONETARY=English_Netherlands.utf8 LC_NUMERIC=C
## [5] LC_TIME=English_Netherlands.utf8
##
## time zone: Europe/Amsterdam
## tzcode source: internal
##
## attached base packages:
## [1] grid      stats     graphics  grDevices datasets  utils     methods
## [8] base
##
## other attached packages:
##  [1] gplots_3.2.0     sigclust_1.1.0.1 diceR_3.1.0      factoextra_1.0.7
##  [5] writexl_1.5.4    VIM_6.2.6        colorspace_2.1-2 lubridate_1.9.4
##  [9] forcats_1.0.1    stringr_1.5.2    dplyr_1.1.4      purrr_1.1.0
## [13] readr_2.1.5      tidyr_1.3.1      tibble_3.3.0     ggplot2_4.0.0
## [17] tidyverse_2.0.0  readxl_1.4.5     pacman_0.5.1
##
## loaded via a namespace (and not attached):
##  [1] tidyselect_1.2.1   farver_2.1.2       S7_0.2.0           bitops_1.0-9
##  [5] fastmap_1.2.0      digest_0.6.37      timechange_0.3.0   lifecycle_1.0.4
##  [9] cluster_2.1.8.1    magrittr_2.0.4     compiler_4.5.1     rlang_1.1.6
## [13] progress_1.2.3     tools_4.5.1        yaml_2.3.10        data.table_1.17.8
## [17] knitr_1.50         labeling_0.4.3     RankAggreg_0.6.6   prettyunits_1.2.0
## [21] sp_2.2-0           mclust_6.1.1       plyr_1.8.9         RColorBrewer_1.1-3
```

```
## [25] abind_1.4-8        KernSmooth_2.23-26 withr_3.0.2        nnet_7.3-20
## [29] caTools_1.18.3     e1071_1.7-16       scales_1.4.0       gtools_3.9.5
## [33] MASS_7.3-65        cli_3.6.5          rmarkdown_2.30     crayon_1.5.3
## [37] generics_0.1.4     rstudioapi_0.17.1  robustbase_0.99-6  reshape2_1.4.4
## [41] tzdb_0.5.0         proxy_0.4-27       clValid_0.7        assertthat_0.2.1
## [45] cellranger_1.1.0   vctrs_0.6.5        boot_1.3-31        Matrix_1.7-3
## [49] carData_3.0-5      car_3.1-3          hms_1.1.4          ggrepel_0.9.6
## [53] Formula_1.2-5      kohonen_3.0.12     clusterCrit_1.3.0  vcd_1.4-13
## [57] glue_1.8.0         DEoptimR_1.1-4     stringi_1.8.7      gtable_0.3.6
## [61] lmtest_0.9-40      pillar_1.11.1      htmltools_0.5.8.1  R6_2.6.1
## [65] evaluate_1.0.5     lattice_0.22-7     renv_0.17.0        class_7.3-23
## [69] Rcpp_1.1.0         laeken_0.5.3       ranger_0.17.0      xfun_0.53
## [73] zoo_1.8-14         pkgconfig_2.0.3
```
